# Psychological experience and coping strategies of patients in the Northeast US delaying care for infertility during the COVID-19 pandemic

**DOI:** 10.1186/s12958-021-00721-4

**Published:** 2021-02-23

**Authors:** David B. Seifer, William D. Petok, Alisha Agrawal, Tanya L. Glenn, Arielle H. Bayer, Barry R. Witt, Blair D. Burgin, Harry J. Lieman

**Affiliations:** 1 Department Obstetrics, Gynecology and Reproductive Sciences, Yale School of Medicine, New Haven, CT USA; 2 Department of Obstetrics and Gynecology, Sidney Kimmel Medical College, Thomas Jefferson University, Philadelphia, PA USA; 3 Department of Psychiatry, Dartmouth Hitchcock Medical Center, Lebanon, New Hampshire USA; 4 Department Obstetrics & Gynecology and Women’s Health, Albert Einstein School of Medicine, Bronx, NY USA; 5 Department of Psychology, La Salle University, Philadelphia, PA USA

## Abstract

**Background:**

On March 17, 2020 an expert ASRM task force recommended the temporary suspension of new, non-urgent fertility treatments during an ongoing world-wide pandemic of Covid-19. We surveyed at the time of resumption of fertility care the psychological experience and coping strategies of patients pausing their care due to Covid-19 and examined which factors were associated and predictive of resilience, anxiety, stress and hopefulness.

**Methods:**

Cross sectional cohort patient survey using an anonymous, self-reported, single time, web-based, HIPPA compliant platform (REDCap). Survey sampled two Northeast academic fertility practices (Yale Medicine Fertility Center in CT and Montefiore’s Institute for Reproductive Medicine and Health in NY). Data from multiple choice and open response questions collected demographic, reproductive history, experience and attitudes about Covid-19, prior infertility treatment, sense of hopefulness and stress, coping strategies for mitigating stress and two validated psychological surveys to assess anxiety (six-item short-form State Trait Anxiety Inventory (STAl-6)) and resilience (10-item Connor-Davidson Resilience Scale, (CD-RISC-10).

**Results:**

Seven hundred thirty-four patients were sent invitations to participate. Two hundred fourteen of 734 (29.2%) completed the survey. Patients reported their fertility journey had been delayed a mean of 10 weeks while 60% had been actively trying to conceive > 1.5 years. The top 5 ranked coping skills from a choice of 19 were establishing a daily routine, going outside regularly, exercising, maintaining social connection via phone, social media or Zoom and continuing to work. Having a history of anxiety (*p* < 0.0001) and having received oral medication as prior infertility treatment (*p* < 0.0001) were associated with lower resilience. Increased hopefulness about having a child at the time of completing the survey (*p* < 0.0001) and higher resilience scores (*p* < 0.0001) were associated with decreased anxiety. Higher reported stress scores (*p* < 0.0001) were associated with increased anxiety. Multiple multivariate regression showed being non-Hispanic black (*p* = 0.035) to be predictive of more resilience while variables predictive of less resilience were being a full-time homemaker (*p* = 0.03), having received oral medication as prior infertility treatment (*p* = 0.003) and having higher scores on the STAI-6 (< 0.0001).

**Conclusions:**

Prior to and in anticipation of further pauses in treatment the clinical staff should consider pretreatment screening for psychological distress and provide referral sources. In addition, utilization of a patient centered approach to care should be employed.

## Introduction

The struggle to conceive with a history of infertility can be psychologically and physically demanding due to both the extensive scheduling and execution of medical testing and treatments (1), and lack of insurance transparency or absence of insurance itself (2). Infertility is also associated with immense psychological stress due to its inability to accomplish a desired social role (3). It presents both as a chronic stressor from the potential loss of a personal ideal as well as an acute stressor which results from both the anticipation and the undergoing of a timely workup and the treatment itself. Trouble conceiving challenges men and women’s hopes of being a parent, expectations for their futures and confidence in one’s body and health (4). The Covid-19 pandemic that began in early 2020 compounded the situation even further by adding more hurdles and delays in fertility treatment. Others have commented on the lack of knowledge about the psychological health of infertile couples during the pandemic (5–7).

Resiliency involves employing strategies that allow one to succeed when faced with adversity, as in the case of a global pandemic, and successfully coping with stress (8). Resilience has been found to be a protective factor that reduces the impact of a myriad of stressors. Importantly, resilience has also been shown to decrease the tendency to experience anxiety in infertility patients (1), as it is well established that anxiety disorders are common in this patient population (9). Other research has demonstrated that resilience is a moderator between infertility related stress and fertility quality of life among Chinese women with infertility (10). We presumed that patients with greater resiliency would have less anxiety and greater coping skills, allowing them to better manage the uncertainty brought on by the impact of COVID-19 on their fertility care. While others have evaluated patient reactions to the suspension of services during the pandemic, most did not employ standardized measures of anxiety and none we found evaluated resiliency with a well validated instrument.

On March 17, 2020, an expert ASRM task force composed of physicians, embryologists and mental health professionals released recommendations for the temporary suspension of new, non-urgent fertility treatments during an ongoing world-wide pandemic. This suspension included new treatment cycles, embryo transfers and elective surgeries. Such recommendations were unprecedented in dealing with an extraordinary crisis which was, at the time, poorly defined, life threatening and ever changing. The lack of warning as well as the ambiguity of such a serious situation led to extreme anxiety on behalf of all involved. From our patients’ perspective the pandemic was disruptive in every aspect of their lives and was perceived as an uncontrollable, poorly understood and unanticipated stressful event. Going forward the issued ASRM recommendations were reviewed and revised by the ASRM task force in two-week intervals based on real time acquired information. This eventually resulted in the recommendation of incrementally reopening clinics as of April 24, 2020.

The purpose of this study was to assess the psychological experience as well as the coping strategies of patients pausing their workup and/or treatment due to the Covid-19 pandemic when fertility services were resumed. Furthermore, we were interested in determining which characteristics were associated with and may be predictive of specific psychological outcomes including anxiety, resilience, stress and hopefulness. This knowledge can be useful for providers who care for fertility patients as the pandemic continues. While fertility clinic closures were a unique event, they are likely to recur or alter the way in which fertility care is delivered in the future (7).

## Materials and methods

This was a cross sectional cohort survey study using an anonymous, self-reported, single time, online, web-based, HIPPA compliant platform (REDCap). The survey sampled two Northeast academic infertility practices (Yale Medicine Fertility Center in New Haven and Greenwich, CT and Montefiore’s Institute for Reproductive Medicine and Health in Hartsdale, NY). Patients who were asked to pause their workup and/or fertility treatment during a period from March 2020 until resumption of care were eligible to participate. Non-English speaking patients were excluded from the study. Upon resumption of care patients received an explanation of the study by either an Epic *My Chart message* or an email and elected to participate or not by taking an online REDcap survey. Patients received one *My Chart* or email reminder 3 days from the initial request. No further attempts were made to send additional reminders to avoid disturbing anyone’s intended sense of privacy or reluctance to participate. Data from multiple choice and open response questions collected demographic, reproductive history, experience and attitudes about Covid-19, infertility treatment, sense of hopefulness, sense of stress, coping strategies for mitigating stress, and to two validated psychological surveys to assess anxiety (six-item short-form State Trait Anxiety Inventory (STAl-6)) and resilience 10-item Connor-Davidson Resilience Scale (CD-RISC-10) with respect to an individual’s delay or pause in evaluation and/or treatment during Covid-19. When appropriate, question responses were answered on a 5-point Likert Scale. Survey questions were created through an iterative process and revised to incorporate changes following a pilot delivery to 3 patients and 5 physicians. Data was collected over a three-month period between 5.22.20 and 8.21.20 after IRB exempt status was granted at both institutions.

### Psychological surveys

#### Six-item short-form State Trait Anxiety Inventory

Speilberger’s State – Trait Anxiety Inventory (STAI) is a sensitive and reliable measure of anxiety but its 40-item length is a barrier to its use. Developed for these circumstances, the STAI-6 has been shown to produce reliable scores comparable to those obtained with the 40-question full-form. It was specifically developed for those situations where the use of the complete form may be contraindicated (11). Other 6-item versions of the STAI have been developed (12) but a comparison study found the Marteau and Bekker version had the best correlation with the original STAI (13). Therefore, the present study employed the Marteau and Bekker version, most recently used with infertility patients being treated with preimplantation genetic testing for aneuploidy (PGT-A) (14).

The STAI questions asked participants to choose the most appropriate statement indicating how they felt, at that moment. Directions indicated that there were no right or wrong answers. Participants were advised not to spend too much time on any one statement but to give the answer that best seemed to describe their present feelings. Each question took the form “I feel …” and respondents could choose “not at all” “somewhat” “moderately so” or “very much so.” Items are scored between 1 and 4. Higher scores on the STAI-6 indicate greater anxiety levels.

#### CD-RISC 10

The Connor Davidson Resilience Scale was initially developed to study posttraumatic stress disorder (PTSD) and the impact of resilience on its treatment. A wide range of studies have developed norms for many populations since its inception. The 10 item version used in this study was developed by Campbell-Sills et al. (15). Normed on 764 individuals in a community sample, it has the advantage of reduced time for completion and a high correlation with the original 25 item CD-RISC. The original survey is a 25-item 5-point Likert-type assessment of “personal qualities that enable one to thrive in the face of adversity” (8). Response categories are “not at all true,” “rarely true,” “sometimes true,” “often true,” and “true nearly all the time,” with responses of “not at all true” worth 0 and “true nearly all the time” worth 4. Participant scores are evaluated based on which quartile they fall into. For the CD-RISC 10 a score of 26 would fall in the lowest 25% of the population, a score of 36 would be the third quartile and 25% of the population would score higher (i.e. 37–40). Scores in the higher quartiles reflect greater levels of resilience. Both the CD-RISC 25 (1) and the CD-RISC 10 (16) have been used with infertility patients.

Participants were asked to “Please indicate how much you agree with the following statements as they apply to you over the last month. If a particular situation has not occurred recently, answer according to how you think you would have felt”. Items took the form: **“**Able to adapt to change”, “Can deal with whatever comes”, “See the humorous side of things”, etc.

### Statistical analysis

R software (R version 3.2.3) (17) was used to conduct the statistical analysis. Survey data including demographics, previous fertility diagnosis/treatment, COVID-19 experience, coping strategies, and STAI-6 and CDRISC-10 scores were summarized for the dataset. Means and standard deviations were calculated for quantitative data and counts and percentages were calculated for qualitative data. Correlations were then calculated between these survey data of demographic and fertility information, COVID-19 experience and coping strategies and our specific outcomes of interest which included CDRISC-10 scores, STAI-6 scores, stress of journey scores and hopefulness about having a child at the time of completion of the survey scores. Kendall rank correlations were calculated between continuous and ordinal variables, ordinal and ordinal variables, and between non-normally distributed continuous variables. Point biserial correlations were calculated between continuous and binary variables while rank biserial correlations were calculated between ordinal and binary variables. Correlations with a *p*-value of < 0.05 with Bonferroni correction for multiple comparisons were considered significant.

Multivariate model selection using Least Absolute Shrinkage and Selection Operator (LASSO) with stability selection was then performed using the R ‘Stabs’ (18) package to determine the most important variables in predicting the outcomes of interest. LASSO is a statistical method that uses regularization techniques for multivariate model selection. It was developed to reduce over-fitting and improve prediction accuracy over traditional stepwise regression statistical techniques. Stability selection further improves the variable selection process by reducing the number of falsely selected noise variables that can occur in high dimensional datasets. Stability selection with LASSO runs several multiple multivariate regression models on subsets of the full dataset and returns the proportion of times each variable was selected for by LASSO (19). For the analysis, we normalized each of the variables and then sorted them by the proportion of times they were selected by LASSO with stability selection. We included the top five variables as predictors in our multivariate models to predict each of our outcomes of interest.

## Results

### Demographic information

A total of 734 patients were sent invitations with a single reminder to consider participation in this study. Of the total invitations sent, 214 out of 734 (29.2%) patients completed the survey. Data were collected in departments of obstetrics and gynecology and patients were therefore female. Table 1 notes that 3 men were in the sample. Subjects were asked how they self-identified with respect to gender. Those 3 subjects who identified themselves as male are transmen who had not undergone gender affirming surgery and were therefore capable of conception. The mean age (+SD) of those responding was 35.5 + 4.1 years old. 56.5% were non-Hispanic white, 16.4% were Hispanic, 9.3% non-Hispanic black. 81.3% were married heterosexual, 6% single living alone, 4.2% same sex married. 88.8% had a bachelor’s degree or higher. 66.8% reported having no children while 27.6% had a single child and 5.2% with at least 2 children. Most reported (81.3%) being employed full time and 38% having an income of a $100,000 or more. Please see Table 1 for summary of demographic profile.

**Table 1:** Demographics of survey participants^a^

	***n*** = 214
Age mean (sd)	35.5(4.1)
Sex n(%)	
*Female*	202(94.4%)
*Male*	3(1.4%)
Race/ethnicity n(%)	
*Non-Hispanic White*	121(56.5%)
*Hispanic*	35(16.4%)
*Non-Hispanic Black*	20(9.3%)
*Asian*	17(7.9%)
*Other*	9(4.2%)
*Prefer not to say*	8(3.7%)
Religion n(%)	
*Christian*	132(61.7%)
*No Religion*	34(15.9%)
*Jewish*	17(7.9%)
*Prefer not to say*	12(5.6%)
*Hindu*	6(2.8%)
*Muslim*	6(2.8%)
*Buddhist*	2(0.9%)
*Other*	1(0.5%)
Relationship Status n(%)	
*Married (Heterosexual)*	174(81.3%)
*Single living alone*	13(6.1%)
*Married (Same sex)*	9(4.2%)
*In a relationship, unmarried*	8(3.7%)
*Single living with heterosexual partner*	7(3.3%)
*Other*	1(0.5%)
What is the highest education level you completed? n(%)	
*Masters Degree*	92(43%)
*Bachelors Degree*	60(28%)
*Professional Degree (PhD, MD, DO, JD,* et al.*)*	38(17.8%)
*High School Graduate or equivalent*	13(6.1%)
*Associates Degree*	9(4.2%)
*Less than High School*	1(0.5%)
Employment n(%)	
*Employed full time*	174(81.3%)
*Employed part time*	16(7.5%)
*Unemployed*	12(5.6%)
*Full-time homemaker*	6(2.8%)
Income n(%)	
*Less than $100,000*	103(48.1%)
*$100,000 or more*	83(38.8%)
*Prefer not to say*	27(12.6%)
How many children do you have? n(%)	
*0*	143(66.8%)
*1*	59(27.6%)
*2*	10(4.7%)
*3*	1(0.5%)

^a^*Note that that the denominator, n = 214, is based on the total number of patients who responded to the survey questions but not all survey questions were required. Totals do not equal 100% in each category as not all patients answered every survey question*

### Fertility history information

Patients reported that their fertility journey was delayed a mean of 10 weeks while 60% had been actively trying to have a baby for more than 1.5 years prior to the delay. 55.6% reported being pregnant before. Among these patients over two-thirds had experienced a previous pregnancy loss. Most patients reported having a confirmed infertility diagnosis (57.5%) with ovarian problems (31%) and unexplained infertility (26%) being the most common diagnosis. Most patients had tried intrauterine insemination (IUI) (37%), oral medications (36%) or in vitro fertilization (IVF) (34%) prior to the delay and 60.3% were considering IVF while 37% were considering frozen embryo transfer (FET) for their next infertility step. The majority of respondents (80.8%) had not taken medications for depression or anxiety in the last 12 months prior to the delay. Three fourths of surveyed patients believed that emotional stress could reduce the success of treatment. Yet, there is little to no rigorous research demonstrating a biological causality for infertility due to emotional stress (20, 21). A group of patients reported they had not paused their treatment (33.6%). Those individuals were between phases of treatment and waiting to identify a donor or gestational carrier and therefore did not experience a pause in treatment. These processes take weeks or months and started before the pandemic and did not end until after restrictions were eased, Hence, no pause was experienced. Please see Table 2 for summary of fertility history.

**Table 2:** Fertility information of survey participants^a^

	***n = 214***
At what point in evaluation or treatment did you pause? n(%)	72(33.6%)
*During fertility testing/evaluation prior to actual fertility treatment*	34(15.9%)
*During non-* In Vitro *Fertilization (IVF) fertility treatment*	30(14%)
*Prior to starting any fertility testing/evaluation*	27(12.6%)
*During IVF treatment after egg retrieval but cancelled transfer (froze embryos)*	21(9.8%)
*During frozen embryo transfer (FET) cycle*	15(7%)
*During IVF treatment prior to egg retrieval*	12(5.6%)
*I have not paused*	72(33.6%)
How many weeks do you believe your fertility journey was delayed? mean (sd)	10(5)
Does your health insurance cover any part of your infertility treatment? n(%)	
*Yes*	175(81.8%)
*No*	33(15.4%)
*Do not know*	4(1.9%)
How long have you been actively trying to have a baby? n(%)	
*> 48 months*	53(24.8%)
*18–24 months*	31(14.5%)
*12–18 months*	29(13.6%)
*36–48 months*	25(11.7%)
*6–12 months*	25(11.7%)
*< 6 months*	22(10.3%)
*24–36 months*	20(9.3%)
How many times have you been pregnant? mean (sd)	1.1(1.4)
Have you had a pregnancy loss? n(%)	
*No*	124(57.9%)
*Yes*	83(38.8%)
How many pregnancy losses have you had? mean (sd)	0.7(1.2)
Do you have a confirmed infertility diagnosis? n(%)	
*Yes*	123(57.5%)
*No*	60(28%)
*Do not Know*	24(11.2%)
Choose up to 2 reasons from the list n(%)	
*Ovulation problem (*i.e. *polycystic ovarian syndrome*	38(17.8%)
*Unexplained*	32(15%)
*Advanced age/ decreased ovarian reserve (low number of eggs)*	26(12.1%)
*Male factor*	25(11.7%)
*Blocked fallopian tubes*	16(7.5%)
*Other*	11(5.1%)
*Endometriosis*	9(4.2%)
*Anatomical uterine problem*	3(1.4%)
*Fibroids*	3(1.4%)
Infertility treatments you have had prior to pausing treatment: n(%)	
*Intrauterine Insemination (IUI)*	79(36.9%)
*Oral medication (Clomid, Letrozole)*	77(36%)
In Vitro *Fertilization (IVF)*	73(34.1%)
*None*	63(29.4%)
*Injectable (Menopur, Gonal-F)*	58(27.1%)
*Frozen Embryo Transfer (FET)*	33(15.4%)
*Egg freezing*	28(13.1%)
*Surgery*	23(10.7%)
*Endometriosis treatment*	6(2.8%)
*Other*	5(2.3%)
*Donor sperm*	3(1.4%)
*Do not know*	2(0.9%)
*Donor eggs*	1(0.5%)
*Gestational Carrier/Surrogate*	1(0.5%)
Infertility treatments you are considering: n(%)	
In Vitro *Fertilization (IVF)*	129(60.3%)
*Frozen Embryo Transfer (FET)*	79(36.9%)
*Injectables (Menopur, Gonal-F)*	51(23.8%)
*Intrauterine Insemination (IUI)*	41(19.2%)
*Oral medication (Clomid, Letrozole)*	39(18.2%)
*Egg freezing*	17(7.9%)
*Donor sperm*	9(4.2%)
*Donor eggs*	8(3.7%)
*Surgery*	7(3.3%)
*Do not know*	5(2.3%)
*Endometriosis treatment*	5(2.3%)
*Gestational Carrier/Surrogate*	4(1.9%)
*None*	4(1.9%)
*Other*	3(1.4%)
Have you taken any medication for anxiety depression (last 12 months)? n(%)	
*No*	173(80.8%)
*Yes*	34(15.9%)
Do you believe that emotional stress: (select all that apply) n(%)	
*Can reduce success of treatment*	162(75.7%)
*Can cause miscarriage*	96(44.9%)
*Can cause infertility*	94(43.9%)
*Has no impact on fertility*	12(5.6%)

^a^*Note that that the denominator, n = 214, is based on the total number of patients who responded to the survey questions but not all survey questions were required. Totals do not equal 100% in each category as not all patients answered every survey question*

### Covid-19 experience and attitudes

Most of the respondents (60.7%) were required to shelter in place for a mean of 9.8 weeks during this pause. Of those patients who were required to shelter in place, 8.5% reported sheltering in place with 0 people, 50% reported sheltering in place with 1 other person, and 40% reported sheltering in place with 2 or more people. Most patients reported working from home (53.3%) during the treatment pause and 72.9% did not have a change in employment. On a scale of 1–5, with 1 being most important and 5 being least important, patients were asked to rank various stressors from most to least important during the treatment pause and currently. During the treatment pause, patients had ranked mental health (2.4) first followed by physical health (2.6), personal safety (3), strain on relationship with partner (3.6), and financial situation (3.7). At time of completing the survey, patients ranked physical health (2.3) first followed by mental health (2.4), personal safety (2.9), strain on relationship with partner (3.8), and financial situation (3.8). Most patients did not develop COVID-19 symptoms (82.2%), did not test positive for COVID-19 (86.4%), and were not hospitalized for COVID-19 (90.2%). Most patients were informed about a pause in their care via phone call from a nurse (26.2%) or physician (17.8%) or through EMR messaging (20.1%). Despite this, most patients (58.4%) did not feel part of the decision-making process. Most patients reported feeling frustrated but understanding of the pause (39.3%). On a scale of 1–5, with 1 being least stressful and 5 being most stressful, patients rated the stress of this pause a 3.4. 38.8% of patients could not compare the stress of the pause to the options listed in the survey however, of those that could, 13.6% compared it to changing jobs, 13.1% thought it was comparable to the illness of a close family member, 9.8% to moving their residence, 8.9% to a pregnancy loss, 4.2% to a death of a close family member while only 0.5% compared it to loss of a child. Most patients reported that their fertility clinic did not offer them mental health support services (56.1%) and most patients did not seek mental health assistance (75.7%). However, of those that did seek mental health assistance, 87.5% found it helpful. Note that that the denominator, *n* = 214, is based on the total number of patients who responded to the survey questions but not all survey questions were required. Totals do not equal 100% in each category as not all patients answered every survey question. Please see Table 3 for summary of Covid-19 experience and attitudes.

**Table 3:** COVID-19 experience of survey participants^a^

	***n = 214***
Were you required to shelter in place? n(%)	
*Yes*	130(60.7%)
*No*	64(29.9%)
How many weeks were you sheltered in place? mean (sd)	9.8(6)
How many people besides you were sheltered in place? mean (sd)	1.5(1.1)
Where were you working primarily during your treatment pause? n(%)	
*At home*	114(53.3%)
*At a workplace outside the home*	61(28.5%)
*I was unemployed*	19(8.9%)
Physical Health - rank during pause mean (sd)	2.6(1.5)
Mental Health - rank during pause mean (sd)	2.4(1.3)
Strain on Relationship - rank during pause mean (sd)	3.6(1.5)
Personal Safety - rank during pause mean (sd)	3(1.3)
Financial Situation - rank during pause mean (sd)	3.7(1.4)
Physical Health - rank currently mean (sd)	2.3(1.4)
Mental Health - rank currently mean (sd)	2.4(1.3)
Strain on Relationship - rank currently mean (sd)	3.8(1.5)
Personal Safety - rank currently mean (sd)	2.9(1.3)
Financial Situation - rank currently mean (sd)	3.8(1.3)
Did you develop CV-19 symptoms during your pause of treatment? n(%)	
*No*	176(82.2%)
*Yes*	19(8.9%)
Did you test positive for CV-19 during your treatment pause? n(%)	
*No*	185(86.4%)
*Yes*	10(4.7%)
Were you hospitalized for CV-19 during your pause of treatment? n(%)	
*No*	193(90.2%)
*Yes*	1(0.5%)
Did your employment change (furloughed, reduced hours, lost job)? n(%)	
*No*	156(72.9%)
*Yes*	39(18.2%)
How did your fertility clinic inform you about changes in your care? n(%)	
*Phone call from nurse*	56(26.2%)
*Message on medical record (EMR) portal*	43(20.1%)
*Phone call from physician*	38(17.8%)
*Email*	19(8.9%)
*I wasn’t informed by the clinic*	17(7.9%)
*Phone call from other*	11(5.1%)
*I did not know there was national guidance*	8(3.7%)
*U.S. Postal Service*	1(0.5%)
Did you feel part of the treatment decision making? n(%)	
*No*	125(58.4%)
*Yes*	68(31.8%)
How did you feel about taking a pause? n(%)	
*frustrated but understood*	84(39.3%)
*understood due to the unknown risks to me and office staff*	44(20.6%)
*understood due to the unknown risks to me*	35(16.4%)
*frustrated/angry but resolved over time*	19(8.9%)
*frustrated and angry which persists today*	8(3.7%)
*other*	4(1.9%)
How stressful was this pause from your fertility journey? mean (sd)	3.4(1.3)
Compare the stress of the pause in your treatment to a previous life event? n(%)	
*none of the above*	83(38.8%)
*changing jobs*	29(13.6%)
*illness of a close family member*	28(13.1%)
*to moving your residence*	21(9.8%)
*pregnancy loss*	19(8.9%)
*death of a close family member*	9(4.2%)
*loss of a child*	1(0.5%)
Did your fertility clinic offer you mental health support services? n(%)	
*No*	120(56.1%)
*Not sure*	56(26.2%)
*Yes*	18(8.4%)
Did you seek mental health assistance while your treatment was delayed? n(%)	
*No*	162(75.7%)
*Yes*	32(15%)
If so, did you find it to be helpful? n(%)	
*Yes*	28(13.1%)
*No*	4(1.9%)

^a^*Note that that the denominator, n = 214, is based on the total number of patients who responded to the survey questions but not all survey questions were required. Totals do*

*not equal 100% in each category as not all patients answered every survey question*

### Coping strategies to mitigate stress

From a list of 19 different coping strategies, patients were asked to select their top 5 coping strategies and rank them from 1 to 5 with 1 being most successful and 5 being least successful. Most patients ranked establishing a daily routine (43.5%), going outside regularly (38.8%), exercising (38.3%), maintaining social connection via phone, social media or Zoom (31.8%) or working (27.6%) as one of their top 5 coping skills. Establishing a daily routine had the highest mean rank (2.2) followed by exercise (2.3) and then going outside regularly (2.4). Patients reported exercising a mean of 3.1 days per week. Most patients reported going outside when weather permitted (77.1%) and reported going outside a mean of 5.2 days per week. Marijuana use increased for a small number of patients (1.9%). However, alcohol use increased for almost one fourth (23.8%). While many patients reported no weight change, a large percentage indicated weight fluctuation of 5 or more pounds (31.8%) Most patients reported that they did not change their mind about the treatment they would pursue (69.6%). The majority of patients did not have to change treatment plans due to financial considerations (76.2%) and most patients reported that they would continue with their original treatment plan (75.7%). If such circumstances were to happen again, many patients (40.2%) reported that they would proceed with treatment if they could regardless of unknown risks. Most patients (59.3%) reported that their partner was their greatest support person during the pause.

On a scale of 1–5 with 1 being least hopeful and 5 being most hopeful, patients were asked to rate their hopefulness about having a child prior to the pandemic and their hopefulness about having a child at the time the survey was completed. The mean hopefulness about having a child prior to the pandemic was 4.1 and the mean hopefulness about having a child at the time the survey was completed was 3.9. Finally, patients were asked to complete two psychometric questionnaires to assess the overall anxiety (STAI-6) and resilience (CD-RISC-10) in this population. The mean anxiety score (STAI score) was 45 (range 20–76.7, SD = 13.7) and the mean resilience score (CDRISC-10 score) was 28.2 (Range 11–40, SD = 5.9). To place these scores in perspective, STAI-6 scores range from 20 to 80 with scores greater than or equal to 45 indicating high anxiety. CDRISC-10 scores range from 0 to 40 with scores less than or equal to 29 in the lowest 25% of the general population for resilience. Note that that the denominator, *n* = 214, is based on the total number of patients who responded to the survey questions but not all survey questions were required. Totals do not equal 100% in each category as not all patients answered every survey question. Please see Table 4 for summary of coping strategies, anxiety, and resilience of survey participants.

**Table 4:** Coping strategies, anxiety, and resilience of survey participants^a^

	***n = 214***
How often were the following coping skills in the top 5: n(%)	
*Established a daily routine*	93(43.5%)
*Making sure I went outdoors regularly*	83(38.8%)
*Exercise*	82(38.3%)
*Maintaining social connection* via *phone/FaceTime/Zoom*	68(31.8%)
*Working*	59(27.6%)
*Watching TV/films*	47(22%)
*Affirmative statements such as we will make it through this...*	45(21%)
*Pets*	44(20.6%)
*Acknowledging what you are grateful for*	41(19.2%)
*Journaling*	37(17.3%)
*Sex with my partner*	26(12.1%)
*Social networking (Facebook* etc.*)*	24(11.2%)
*Reading*	23(10.7%)
*Online support group*	20(9.3%)
*Devotional activities (*i.e. *prayer)*	18(8.4%)
*Gardening*	18(8.4%)
*Meditation*	18(8.4%)
*Listening to music*	16(7.5%)
*Other*	5(2.3%)
Established a daily routine rank mean (sd)	2.2(1.3)
Exercise rank mean (sd)	2.3(1.1)
Making sure I went outdoors regularly rank mean (sd)	2.4(1.2)
Pets rank mean (sd)	2.5(1.2)
Affirmative statements rank mean (sd)	2.6(1.2)
Maintaining social connection rank mean (sd)	2.6(1.3)
Devotional activities (i.e. prayer) rank mean (sd)	2.6(1.7)
Listening to music rank mean (sd)	3.1(1.3)
Acknowledging what you are grateful for rank mean (sd)	3.2(1.5)
Other rank mean (sd)	3.2(1.5)
Working rank mean (sd)	3.3(1.3)
Watching TV/films rank mean (sd)	3.5(1.2)
Reading rank mean (sd)	3.5(1.3)
Sex with my partner rank mean (sd)	3.5(1.4)
Gardening rank mean (sd)	3.6(1.3)
Meditation rank mean (sd)	3.7(1.1)
Social networking (Facebook etc.) rank mean (sd)	3.8(1.3)
Journaling rank mean (sd)	4.1(1.2)
Online support group rank mean (sd)	4.2(1.2)
How many days per week did you exercise? mean (sd)	3.1(1.9)
Did you go outside if weather permitted? n(%)	
*Yes*	165(77.1%)
*No*	12(5.6%)
How many times per week did you go outside? mean (sd)	5.2(3.2)
Did you increase use of marijuana? n(%)	
*I do not smoke marijuana*	156(72.9%)
*No*	14(6.5%)
*Yes*	4(1.9%)
Did you increase use of alcohol? n(%)	
*No*	68(31.8%)
*I do not drink alcohol*	54(25.2%)
*Yes*	51(23.8%)
Did your weight go up or down more than 5 lbs.? n(%)	
*No*	108(50.5%)
*Yes*	68(31.8%)
Weight change (lbs.) mean (sd)	3.5(5)
Did you change your mind about the treatment you would pursue? n(%)	
*No*	149(69.6%)
*Yes*	27(12.6%)
Did your treatment plans change because of financial considerations? n(%)	
*No*	163(76.2%)
*Yes*	12(5.6%)
Who was your greatest support person during this pause? n(%)	
*partner*	127(59.3%)
*family member*	30(14%)
*self*	10(4.7%)
*friend*	8(3.7%)
*co-worker*	2(0.9%)
*therapist*	2(0.9%)
What are you going to do now? n(%)	
*Continue with my original treatment plan*	162(75.7%)
*Move to another fertility clinic*	8(3.7%)
*Postpone treatment*	8(3.7%)
How hopeful were you about having a child before the pandemic? mean (sd)	4.1(1.1)
How hopeful are you about having a child now? mean (sd)	3.9(1.2)
If such circumstances happen again, would you: n(%)	
*proceed with treatment if you could regardless of unknown risk*	86(40.2%)
*undecided*	49(22.9%)
*have a pause of several months to stay safe*	43(20.1%)
STAI Score mean (sd)	45(13.7)
CDRISC 10 Score mean (sd)	28.2(5.9)

^a^*Note that that the denominator, n = 214, is based on the total number of patients who responded to the survey questions but not all survey questions were required. Totals do*

*not equal 100% in each category as not all patients answered every survey question*

### Correlations

#### Anxiety

The variables associated with decreased anxiety included increased hopefulness about having a child at the time of completing the survey (*p* < 0.0001) and higher resilience scores (*p* < 0.0001). The variable associated with increased anxiety included higher reported stress (*p* < 0.0001).

#### Resilience

The variables associated with lower resilience included higher anxiety (*p* < 0.0001) and having received oral medication as prior infertility treatment (*p* < 0.0001). No variables were statistically significantly associated with higher resilience after applying the Bonferroni correction for multiple comparisons.

#### Stress

The variables associated with a less stressful journey included being understanding of the pause due to unknown risks (*p* < 0.0001), feeling part of the decision making process (*p* < 0.0001), choosing to have a pause of several months to stay safe if circumstances were to happen again (*p* < 0.0001), not comparing the pause to a previous life event (*p* < 0.0001), not having a pause in treatment (*p* < 0.0001), and not going to therapy for mental health (*p* < 0.0001). The variables associated with a more stressful journey included not feeling part of the decision-making process (*p* < 0.0001), having a higher anxiety score (*p* < 0.0001), having persistent feelings of frustration and anger about the pause (*p* < 0.0001), comparing the pause to a pregnancy loss (*p* < 0.0001), and having an increased delay in treatment (*p* < 0.0001).

#### Hopefulness

The variable associated with more hopefulness about having a child at the time of completing the survey included increased hopefulness about having a child prior to the pandemic (*p* < 0.0001). The variables associated with less hopefulness about having a child now include increased anxiety (*p* < 0.0001) and having to change treatment plans due to financial considerations (*p* < 0.0001).

### Multivariate analysis to predict anxiety

In the multivariate model the variables predictive of less anxiety included higher resilience scores (*p* < 0.0001) and understanding reasons for the pause (*p* = 0.003). The variables predictive of increased anxiety included living alone (*p* = 0.001) and having a confirmed infertility diagnosis (*p* = 0.001).

### Multivariate analysis to predict resilience

In the model, the variables predictive of more resilience included being Non-Hispanic Black (*p* = 0.035). The variables predictive of less resilience included being a full-time homemaker (*p* = 0.03), having received oral medication as prior infertility treatment (*p* = 0.003) and having higher anxiety scores on the STAI-6 Score (< 0.0001). Please see Table 5 for summary of multivariate analysis for psychological outcomes of interest.

**Table 5:** Multivariate analysis on outcomes of interest

Outcome	Predictors	Stability	Coefficient	***p***-Value
STAI Score (Anxiety)	Relationship Status - Single living alone	.55	1.02	.0001*
	Has confirmed infertility diagnosis - Yes	.46	.51	.0001*
	Initial feelings about taking a pause -understood due to the unknown risks to me and/or staff	.49	−.44	.0003*
	CDRISC 10 Score (Resilience)	.86	−.31	<.0001*
	Considering IUI as infertility treatment	.47	.29	.123
CDRISC-10 Score (Resilience)	Employment – Full-time homemaker	.48	−1.01	.03*
	Race/ethnicity – Non-Hispanic Black	.32	.65	.035*
	Location of work – I was unemployed	.39	.51	.06
	Had oral medication as prior infertility treatment	.79	−.48	.003*
	STAI Score (Anxiety)	.85	−.31	<.0001*
Stress of Journey Score	Point in evaluation that treatment was paused - I have not paused	.78	−.95	.003*
	Initial feelings about taking a pause - understood due to the unknown risks to me and/or office staff	.95	−.55	<.0001*
	Felt part of treatment decision making process - Yes	.77	−.34	.036*
	STAI Score (Anxiety)	.92	.28	<.0001*
	Number of weeks patient believes fertility journey was delayed	.95	.26	<.0001*
Hopefulness about having a child now	Therapy - sought therapy and did not help	.58	−1.41	.002*
	Changed treatment plans due to financial considerations	.76	−.93	.001*
	Point in evaluation that treatment was paused - During IVF treatment prior to egg retrieval	.32	−.52	.042*
	Hopefulness about having a child prior to pandemic	.99	.41	<.0001*
	STAI Score (Anxiety)	.99	−.32	<.0001*

### Multivariate analysis to predict stress of journey

In the model, the variables predictive of a less stressful journey were not having a pause in treatment (*p* = 0.003), being understanding of the pause due to the unknown risks (*p* < 0.0001) and feeling part of the decision-making process (*p* = 0.036). The variables predictive of a more stressful journey include a greater number of weeks delayed in the fertility journey (*p* < 0.0001) and higher anxiety scores (*p* < 0.0001).

### Multivariate analysis to predict hopefulness about having a child at the time of completing survey

In the multivariate analysis model, the variable predictive of more hopefulness about having a child at time of completing the survey was being more hopeful about having a child prior to the pandemic (*p* < 0.0001). The variables associated with less hopefulness included going to mental health therapy and it not helping (*p* = 0.002), changing treatment plans due to financial considerations (*p* < 0.001), pausing during IVF treatment prior to egg retrieval (*p* = 0.042) and having higher anxiety scores (*p* < 0.0001).

## Discussion

This study surveys women from the Northeast US who were delayed in seeking infertility care in response to the ASRM guidelines surrounding the onset of the Covid-19 pandemic. Participants had been engaged in their workup or treatment at the time their care was paused. This study is unique in that the participants were surveyed regarding their psychological experience and coping strategies once they had resumed their care and not during their pause when the timetable for the resumption of care would have been uncertain and poorly defined. Furthermore, this study is novel in that it documents preferred strategies of coping by a group of women who were deferred in their fertility care due to the onset of a pandemic as well as highlights characteristics associated with and predictive of psychological outcomes of resilience, anxiety, stress and hopefulness.

The response rate of 29.2% was comparable to previous response rates reported on this topic ranging between 17 and 57% (22–24). Other studies utilizing social media recruiting strategies have reported higher response rates (5, 25). Our survey only contained de-identified data and for this reason it was not possible to follow up on those who did not respond. No financial incentive had been provided or social media employed to incentivize participation in this study. Higher response rates have been noted in those who use financial incentives, social media, or a combination of social media and clinic recruitment (5–7). We acknowledge that a relatively low response rate of 29.2% introduces the potential of selection bias by which patients who chose to fill out the survey may have been different than those that did not and thus may limit the generalizability of the findings. However, this limitation exists for the vast majority of surveys that have been published on this challenging subject and thus must be taken into consideration when reviewing such studies until prospective ones are conducted.

The demographic profile of the surveyed group included respondents from the Northeast with a mean age in their mid-thirties, heterogeneous in race/ethnicity, well-educated, mostly coupled with a partner without any children who were fully employed and moderately affluent. These demographics are probably representative of most infertility practices in urban regions of the Northeast and therefore are a limited sample with respect to the entire US as well as having been disproportionately affected by COVID cases in the initial few months when the pause was in effect. These patients may have also been struggling with other stressors (including job impacts, illness in loved ones) pertinent to locations with higher COVID rates and therefore this may have contributed to a lower response rate. The participants reported a mean delay of 10 weeks after having been trying to conceive for a mean of 1.5 years. For those who had undergone previous treatment about a third were treated with IUI, a third with oral fertility medications and a third with assisted reproductive technology (ART) prior to their delay in treatment.

The mean anxiety score (STAI-6 score) was consistent with a state of high anxiety while the mean resilience score (CDRISC-10) indicated the lowest 25% of the general population for resilience at the time of resuming care. Esposito et al. (5) found a mean STAI score of 49.8 which was consistent with our study’s findings. They had five top major concerns during the pause. These were in order of importance from most to least: mental health, physical health, personal safety, strain on their relationship with their partner and concern regarding their financial situation. These same concerns remained after the pause and upon resumption of care, but physical health became the first concern, while mental health was the second concern. It is speculated that during the delay the stress of not knowing when infertility care would resume accompanied by the additional stresses of daily living during the early phases of Covid − 19 resulted in the primary concern for participants’ mental health. Once fertility care resumed with likely increase in limited activity outside the home, concern over physical health took priority for participants. Those that could relate the stress of the pause that they were feeling after resumption of their care to a different life event compared it to changing jobs (> 13%), illness of a close family member (> 13%), moving their residence (< 10%) or having suffered a pregnancy loss (< 10%). The intensity of their responses seemed to have been less compared to those reported from an infertility practice at Columbia in New York City during the worse part of the pandemic (23). Most likely this difference in perceived intensity was due to the difference in timing that each study made their inquiries ie. early beginning of pandemic in New York City (23) versus our current study after the resumption of care months later during the pandemic.

The five most preferred coping strategies that may have helped in mitigating stress and anxiety included establishing a daily routine, going outside regularly, exercising, maintaining a social connection via phone, social media or Zoom or working from home. Each of these coping strategies offered psychological benefits which have been elaborated elsewhere. Hou et al. (26) have discussed the value of primary routines such as work, maintaining hygiene and sleep as well as secondary routines such as exercise and social interaction, on mental health during periods of acute stress.

High anxiety scores and previous history of receiving oral medications correlated with having less resilience at the time of resuming fertility care. Expectedly, anxiety and resilience showed a negative correlation with one another. Participants were sheltered in place for a mean of 9.8 weeks and a delay in their resumption of care for a mean of 10 weeks. Our findings were consistent with those of other investigators. While quarantine (shelter in place) can be a necessary preventative measure during an infectious disease outbreak, it is often associated with a negative psychological impact (27). According to data published by Ben-Kimhy et al. (24), Covid-19 has introduced new stressors and levels of anxiety for infertility patients. Closures of clinics, which resulted in deferred fertility treatments, led to a sharp increase in anxiety and depression among patients undergoing IVF (24). Many women in their mid-thirties are acutely aware that their fertility and the opportunity for treatment is time sensitive. As a result, the indefinite suspension of fertility treatment can have a large emotional, psychological, and financial impact on these patients (23).

Women were more likely to be less stressed at the time of completing the survey if they tended to be optimistic and resilient about their reproductive future. Decreased anxiety was associated with increased hopefulness about having a child and higher resilience scores while increased anxiety was associated with higher reported stress scores. Variables associated with a more stressful journey included not feeling part of the decision-making process, having a higher anxiety score, having persistent feelings of frustration and anger about the pause, comparing the pause to a pregnancy loss and having an increased delay in treatment. Increased anxiety and having to change treatment plans due to financial considerations were associated with less hopefulness about having a child at the time of completing the survey.

A racial designation of Non-Hispanic Black was predictive of more resilience. There is some research to suggest that Black Americans may be more resilient than white Americans (28). As Assari notes “… lack of preparedness and experience with previous stressors may place whites at the highest risk of poor outcomes when life gets out of control. Minority groups, on the other hand, have consistently lived under economic and social adversities which has given them firsthand experience and ability to believe that they can handle the new stressors. For blacks a stressor is anything but new. They have mastered their coping skills.” COVID-19 has been likened to life getting out of control.

It has been shown that anxiety is the biggest psychological obstacle for infertile patients (29). However, social support is an important factor in mitigating the experience of anxiety. Personal resources work as a protective factor in times of crisis and aid in reducing levels of distress (24). It has been noted that stress from infertility decreases as resources and strategies related to social coping increase (30). Women with infertility experience more anxiety when they perceive low levels of social support from their partners and other family members (29). Also, individuals with higher levels of resilience are better equipped to actively apply social coping methods (31). Closures of clinics, which resulted in deferred fertility treatments, can lead to an increase in anxiety and depression among patients undergoing IVF (24). For many, fertility treatments are time sensitive therefore, the indefinite suspension of fertility treatment can have a large emotional, psychological, and financial impact on patients (23).

Alvord and Grados (32) have noted that resiliency is a set of competencies and skills that can be learned. Others have noted that the availability of community support systems outside the family can bolster resilience (33). They note that resilience training can be preventative for both healthy and at-risk populations as well as therapeutic for those with clinical symptoms. Fertility clinics with mental health providers embedded in them can provide such training or refer out to providers with the requisite knowledge to work with the infertility population.

Based on the findings of this survey and others (5–7) it is likely that additional waves of this or other pandemics will interrupt service delivery. Prior to and in anticipation of a pause in treatment the clinical staff should consider the following. Employ pretreatment screening for psychological distress and provide referral sources (34). Utilize a patient centered approach to care (22, 35, 36). Approach each patient in the most reassuring manner possible while acknowledging that anxiety and stress are normal reactions to this type of situation. Include a discussion about resuming care as soon as clinical staff is convinced it is safe for patients to resume to minimize the indefiniteness of the situation. When possible, clinical staff should allow patients to be part of the decision-making process. A mention of coping skills that might be beneficial and address their potential mental or physical health concerns during the upcoming pause in care may mitigate their stress as they await a resumption of their care. Suggested coping skills might include establishing a daily routine, going outside regularly, exercising, maintaining a social connection via phone, social media or Zoom and/or working from home or anything they are aware of that was useful to them during their previous experience with the pandemic. Suggest, if patient is in a significant relationship, that leaning on each other’s partner during this crisis may be beneficial.

## Conclusions

We conducted a patient survey of two Northeast academic fertility practices in the US at the time of resumption of fertility care to assess the psychological experience and coping strategies of patients pausing their care due to Covid-19. We determined specific factors that were associated and predictive of resilience, anxiety, stress and hopefulness.

The top 5 ranked coping skills from a choice of 19 were establishing a daily routine, going outside regularly, exercising, maintaining social connection via phone, social media or Zoom and continuing to work. Having a history of anxiety and having received oral medication as prior infertility treatment were associated with lower resilience. Increased hopefulness about having a child at the time of completing the survey and higher resilience scores were associated with decreased anxiety. Higher reported stress scores were associated with increased anxiety. Multiple multivariate regression showed being non-Hispanic black to be predictive of more resilience while variables predictive of less resilience were being a full-time homemaker, having received oral medication as prior infertility treatment and having higher scores on the STAI-6.

Prior to and in anticipation of further pauses in treatment the clinical staff should employ pretreatment screening for psychological distress and provide referral sources. In addition, utilization of patient centered approach to care should be considered. Other recommendations to mitigate stress are described.

## Data Availability

The datasets during and/or analysed during the current study available from the corresponding author on reasonable request.

## Abbreviations

ASRM: American Society for Reproductive Medicine
STAl-6: Six-item short-form State Trait Anxiety Inventory
CD-RISC-10: 10-item Connor-Davidson Resilience Scale
IRB: Institutional Review Board
PGT-A: Preimplantation genetic testing for aneuploidy
LASSO: Least Absolute Shrinkage and Selection Operator
IUI: Intrauterine insemination
IVF: In vitro fertilization
FET: Frozen embryo transfer
