# Correction to: Psychological experience and coping strategies of patients in the Northeast US delaying care for infertility during the COVID-19 pandemic

**DOI:** 10.1186/s12958-021-00746-9

**Published:** 2021-04-28

**Authors:** David B. Seifer, William D. Petok, Alisha Agrawal, Tanya L. Glenn, Arielle H. Bayer, Barry R. Witt, Blair D. Burgin, Harry J. Lieman

**Affiliations:** 1 Department Obstetrics, Gynecology and Reproductive Sciences, Yale School of Medicine, New Haven, CT USA; 2 Department of Obstetrics and Gynecology, Sidney Kimmel Medical College, Thomas Jefferson University, Philadelphia, PA USA; 3 Department of Psychiatry, Dartmouth Hitchcock Medical Center, Lebanon, New Hampshire USA; 4 Department Obstetrics & Gynecology and Women’s Health, Albert Einstein School of Medicine, Bronx, NY USA; 5 Department of Psychology, La Salle University, Philadelphia, PA USA

**Correction to: Reprod Biol Endocrinol 19, 28 (2021)**

**https://doi.org/10.1186/s12958-021-00721-4**

Following publication of the original article [1], the authors reported errors in in the main text.

Abstract, Methods

“Cross sectional cohort patient survey using an anonymous, self-reported, single time, web-based, HIPPA….” Should read “Cross sectional cohort patient survey using an anonymous, self-reported, single time, web-based, *HIPAA…”*

Introduction, 2nd paragraph, 2nd line “due to the Covid-19…” should read “due to the *COVID-19*…”

Materials and Methods, 8th and 11th lines “Covid-19” should read “COVID-19”

Heading Covid-19 experience and attitudes, should read COVID-19

Last sentence of that section “Please see Table 3 for summary of Covid-19 experience and attitudes” should read “Please see Table 3 for summary of COVID-19 experience and attitudes.”

Discussion section, 1st paragraph 2nd sentence: “of the Covid-19 pandemic” should read “of the COVID-19 pandemic”

Conclusions section, 1st paragraph, 2nd sentence “psychological experience and coping strategies of patients pausing their care due to Covid-19.” Should read “psychological experience and coping strategies of patients pausing their care due to COVID-19. “
